# A novel deletion in proximal 22q associated with cardiac septal defects and microcephaly: a case report

**DOI:** 10.1186/1755-8166-2-9

**Published:** 2009-02-24

**Authors:** Caroline Mackie Ogilvie, Joo Wook Ahn, Kathy Mann, Roland G Roberts, Frances Flinter

**Affiliations:** 1 Cytogenetics Department, Guy's & St Thomas' NHS Foundation Trust, London, UK; 2 Department of Medical & Molecular Genetics, King's College London School of Medicine, London, UK; 3 Genetics Department, Guy's & St Thomas' NHS Foundation Trust, London, UK

## Abstract

**Background:**

Proximal 22q is rich in low copy repeats (LCRs) which mediate non-allelic homologous recombination and give rise to deletions and duplications of varying size depending on which LCRs are involved.

**Methods:**

A child with multiple septal defects and other congenital anomalies was investigated for genome imbalance using multiplex ligation-dependent probe amplification (MLPA) for subtelomeres and microdeletion loci, followed by array comparative genomic hybridization (CGH) using oligonucleotide arrays with 44,000 probes across the genome.

**Results:**

MLPA identified a single probe deletion in the SNAP29 gene within band 22q11.21. Follow-up array CGH testing revealed a ~1.4-Mb deletion from 19,405,375 bp to 20,797,502 bp, encompassing 28 genes.

**Conclusion:**

This deletion is likely to be causally associated with the proband's congenital anomalies. Previous publications describing deletions in proximal 22q have reported deletions between LCRs 1 to 4, associated with 22q11 deletion syndrome; in addition, deletions between LCRs 4 and 6 have been described associated with "distal 22q11 deletion syndrome". To our knowledge, this is the first deletion which spans LCR4 and is not apparently mediated by LCRs. Comparison of the phenotypes found in conjunction with previously reported deletions, together with the function and expression patterns of genes in the deleted region reported here, suggests that haploinsufficiency for the Crk-like (CRKL) gene may be responsible for the reported cardiac abnormalities.

## Background

The proximal region of the chromosome 22 long arm is rich in low copy repeats (LCRs), which are known to mediate non-allelic homologous recombination (NAHR)[1]. The most common of these events is the recombination between LCRs 2 and 4, which gives rise to a 3-megabase (Mb) deletion, associated with chromosome 22q11 deletion syndrome (which includes features of Velocardiofacial syndrome and DiGeorge syndrome)[2]. The reciprocal duplication event gives rise to milder but similar features[3,4]. Until recently, these reciprocal events were thought to be underdetected, as theoretically they should occur at the same frequency as the deletion. However, Turner et al have shown, based on modeling of the deletion/duplication, that the deletion events are likely to be more prevalent[5]. The 1.5-Mb deletion that occurs following NAHR between LCRs 2 and 3 is associated with similar features to those found in individuals with the 3-Mb deletion[6].

A number of studies of individuals with dysmorphism and/or developmental delay has revealed a cluster of deletions distal to LCR4, the distal end of the common 3 Mb deletion [7-10]. The patients described in these papers have a range of phenotypic features, which are not found associated with the more proximal deletions. In this paper we describe a patient with an atypical deletion spanning LCR4, and compare her features with those found in overlapping deletions.

## Case presentation

### Clinical details

The patient is the first child born in this relationship to healthy non-consanguineous parents and her father has an older child who is healthy. Her mother (150 cm in height) is Caucasian and her father (180 cm) is half Maltese.

During the pregnancy, bowel problems were suspected after a routine week 13 ultrasound scan was abnormal. She was delivered at 36 and a half weeks by lower segment cesarean section in response to her failure to progress; her birth weight was 1.76 kg with symmetrical growth retardation, and a head circumference of 28.5 cm At birth she was found to have multiple septal defects (both atrial and ventricular – ASDs and VSDs), two of which have been patched surgically and two are being observed with the expectation that they will close spontaneously. She had an imperforate anus (with no fistula) requiring a colostomy, which was reversed when she was 18 months old. Apart from microcephaly, brachycephaly and slight clinodactyly of the right 5^th ^finger she did not have any dysmorphic features and there was no evidence of asymmetry. Her length was first measured at 17 weeks (13 weeks corrected for prematurity) and was 54 cm.

Postnatally she continued to demonstrate growth failure, with height and weight consistently on or just below the 0.4^th ^centile, and her head circumference is growing well below the 0.4^th ^centile. The relative proportions of her head circumference, height and weight have not changed with time. Other areas of development are causing no concerns.

## Methods

G-banded chromosome analysis was carried out on cultured peripheral lymphocytes, using standard laboratory techniques. Multiplex ligation-dependent probe amplification (MLPA; MRC Holland) was carried out using kits for the subtelomere regions of every chromosome (P036B and P069), and a kit for the common microdeletion loci (P245). Array comparative genomic hybridization (array CGH) used oligonucleotide arrays containing approximately 44,000 probes across the genome (Agilent). The proband's DNA was labeled with two different fluorochromes, then co-hybridized with two other patients' DNA, differentially labeled, to give a "dye swap" and hence added confidence in the results.

## Results

Standard karyotype analysis and MLPA testing with probes for the subtelomere regions did not detect any abnormality. However, MLPA with probes for the common microdeletion loci revealed an apparent deletion in the SNAP29 gene. This gene lies at the distal end of the common 3-Mb 22q11 deletion interval. All other, more proximal, probes in this region showed normal copy number. MLPA testing of both parents showed that this finding was *de novo *in the proband, and therefore unlikely to be caused by an inherited single nucleotide polymorphism (SNP) at the ligation site of the MLPA probe. Although a balanced insertional translocation in one of the parents has not been specifically excluded, it is considered most likely that the deletion in the proband arose as a result of crossing over between LCRs (see below), the mechanism for submicroscopic interstitial deletions in most cases.

**Figure 1:**
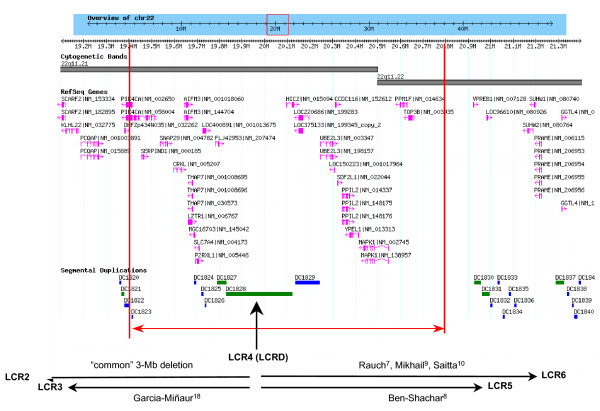
**Deletion breakpoints and the genomic region around the deletion**.

Array CGH was therefore carried out and showed an approximately 1.5-Mb deletion (minimum 19,405,634 – 20,797,302 and maximum 19,288,984 – 20,834,070 bp from the chromosome 22 short arm telomere), containing approximately 28 genes. Figure 1 shows the deletion breakpoints and the genomic region around the deletion.

## Discussion

Previous papers have described "atypical" deletions distal to the common 3-Mb deletion associated with DiGeorge/Velocardiofacial syndrome [7-10] (see Figure 1). The patients described in these papers have relatively mild phenotypes, with few common features. The deletions described all end within LCRs, which are thought to mediate the deletion events via NAHR. A further paper[11] describes a patient with a deletion apparently extending from within the common 3-Mb deletion interval to a locus distal to LCR4. However, close inspection of the data reveals that there are no informative markers beyond LCR4 for this patient, and the published figure in fact represents the maximum deleted region. There is no evidence for deletion beyond LCR4 for this patient.

The patient described in the current work is apparently unique in having a deletion which spans LCR4, the distal point of the common deleted region, and the proximal point of many of the "atypical" distal deletions. Her phenotype may therefore shed light on the relative role of the genes around LCR4 in contributing to the features associated with these deletions. The breakpoints of this deletion do not correspond to any of the major LCRs found on chromosome 22. However, we have identified a 3.7-kb block of high (97%) similarity between the two breakpoint regions – this is 19,351,886–19,355,637 in the proximal breakpoint region and 20,799,044–20,802,748 in the distal breakpoint region. This homology may provide the mechanism for this novel deletion.

Table 1 shows the phenotypic features found in the published cases, and in the case described here. Only cardiac defects and other features found in more than one individual are included.

**Table 1:** Phenotype of patients with "atypical" 22q11 distal deletions

	Garcia-Minaur^18^ LCR30–LCR4 ~1.5 Mb microsats/FISH 1 case (pat)	Our case ~1.4 Mb oligo array	Ben Shachar^8^ LCR4–LCR5 ~1.4 Mb BAC arrays 4 Cases	Saitta^10^ LCR4–LCR6 ~2.1 Mb cosmid FISH 1 case	Ben Shachar^8^ LCR4–LCR6 ~2.1 Mb BAC arrays 2 Cases	Rauch^7^ LCR4–LCR6 ~2.1 Mb FISH/PCR 1 case	Mikhail^9^ LCR4–LCR6 1.55–1.92 Mb BAC arrays 1 case
Cardiac defect	TOF	VSD+ASD	1/4 bicuspid aortic valve	VSD+ truncus arteriosus	1-/2 truncus arteriosus	VSD	-
bifid uvula				+			
cleft palate			-		1/2		
short stature		+		+			
microcephaly		+		+			
hypoplastic nasal alae			3/4	+	1/2		
smooth philtrum			+	+	+		
dev delay			3/4		+		
arched eyebrows			2/4		+		+

This region contains 28 obvious gene-like items (see Additional file 1). Of these, the following are either pseudogenes or are one of several copies – MGC16703 (tubulin pseudogene), RBP3.2, RBP3.3 (two of the three copies are deleted here), PI4KAP2 (probable pseudogene of PIKA4CA, also deleted here), GGT2 (primate-specific duplication of GGT1). This leaves 23, of which 21 are protein-coding and 2 are miRNA-encoding. Of these, three are unlikely candidates for the cardiac anomalies, as there are reported knock-out mice with no reported cardiac phenotype or heterozygote phenotype (UBE2L3[12], MAPK1/ERK2[13], TOP3B[14]) and one (SNAP29) is unlikely because null mutations in humans yield a recessive condition with no cardiac component[15]. Other genes range from known functions which are hard to connect with the phenotype, to completely unknown function; only circumstantial considerations (e.g. expression profiles, often in adult tissues) can be used to rule them in or out.

The strongest candidate, however, is CRKL. This has a highly relevant knock-out mouse phenotype: crkl-/- mice die in utero with cranial nerve and aortic defects and 100% have VSD with overriding aortic arch, and may also have defective thymus, thyroid and parathyroid[16]. The heterozygote mouse (crkl+/-) only has craniofacial and/or thymic defects, but when combined with heterozygosity for tbx1, DGS-like heart defects are seen[17]. Haploinsufficient effects are notoriously strain- and species-dependent, so it seems likely that the CRKL+/- state in humans could give some of the defects seen in the crkl+/-;tbx1+/- mouse, which could be quite variable between individuals. The heart defects seen in our patient may therefore be due to haploinsufficiency of this gene; the position of the CRKL gene proximal to LCR4 means that our patient's cardiac defects could be regarded as a subset of the defects found in "classic DGS". As there are no consistent phenotypic differences between LCR2-4 and LCR2-3 DGS cases, the effects of CRKL+/-, although substantial on their own, may be masked by TBX1+/-. Garcia-Miñaur et al[18] in their paper describing a LCR3-4 deletion, also suggest CRKL as a candidate gene for the cardiac defects found in their patient. The deletion interval we describe thus provides a tighter focus on this gene, with the caveat that our patient shows only septal defects, rather than the Tetralogy of Fallot found by Garcia-Miñaur.

Table 1 shows that cardiac defects have been found in 4 other individuals who have more distal deletions, and are therefore not haploinsufficient for CRKL, indicating that there are likely to be other cardiac-related genes distal to LCR4. However, in the absence of obvious candidates for these genes in the region of overlap between our patient and the patients with deletions distal to LCR4, these other genes are likely to lie beyond the distal breakpoint in our patient. Apart from growth retardation leading to short stature and disproportionate microcephaly, and very minor dysmorphisms, there are no features shared by our patient and the others described with deletions distal to LCR4. The genes in the common deletion interval may therefore be copy number-independent, or have variable penetrance.

## Conclusion

In conclusion, although the possibility remains that one or more of the genes in our patient's deleted interval distal to LCR4 may be responsible for her cardiac defects, this case has provided evidence for a role for haploinsufficiency of CRKL in abnormalities of cardiac development. Further studies investigating the status of this gene in patients with microcephaly in association with septal defects or Tetralogy of Fallot may provide further evidence of this causal link.

## Consent

Written informed consent was obtained from the patient's family for publication of this case report. A copy of the written consent is available for review by the Editor-in-Chief of this journal

## Competing interests

The authors declare that they have no competing interests.

## Authors' contributions

CMO led the laboratory investigations and wrote the manuscript. JWA analysed and interpreted the MLPA and array CGH data. KM analysed and interpreted the MLPA and array CGH data. RR researched the function of the genes in the deletion interval and co-wrote the manuscript. FF initiated the clinical and laboratory investigations of the patient, and provided the phenotype information.

## Supplementary Material

Additional file 1 **Table 2**. 28 obvious gene-like items. Click here for file
