# Supplementary material for: A novel deletion in proximal 22q associated with cardiac septal defects and microcephaly: a case report
Source: Mol Cytogenet. 2009 Feb 24;2:9. doi: 10.1186/1755-8166-2-9 (PMC2669095; doi:10.1186/1755-8166-2-9)
Supplement: Additional file 1 — Table 2. 28 obvious gene-like items. [file 1755-8166-2-9-S1.doc]

| Locus name | Description | Domain structure, likely function | Expression | Disease association | Reference (PMID) | Candidacy |
| --- | --- | --- | --- | --- | --- | --- |
| PIKA4CA | PtdIns 4-kinase alpha | Phosphorylates lipid inositol headgroups |  | Association with schizophrenia |  |  |
| SERPIND1 | Serpin D1/Heparin cofactor 2 precursor (HC-II)/Leuserpin-2 (HLS2) | Protease inhibitor (coagulation inhibitor of thrombin) |  |  | Law et al^1^ | Unlikely to have heterozygous phenotype. Probably clotting defect? |
| SNAP29 | Synaptosomal associated protein 29/soluble NSF attachment protein | Vesicle fusion (t-SNARE) |  | Mutated in CEDNIK syndrome (homozygous LOF mutation) - skin & CNS involvement | Sprecher et al^2^ | No cardiac phenotype; no heterozygous phenotype |
| CRKL | Crk-like | SH2 & SH3 containing proto-oncogene | Ubiquitous, high in pharynx, neural, neural crest | Mouse KO +/- normal, but some have craniofacial/thymic problems. Combines with Tbx1+/- to give DGS-like. Crkl-/- very DGS-like | Guris et al^3, 4^ | Some heterozygous phenotype in mouse. Homozygote has heart defects. Implicated in DGS |
| AIFM3 | Apoptosis-inducing factor 3/AIFL | Rieske iron & FAD-dependent reductase. | Ubiquitous, mitochondria |  | Xie et al^5^ |  |
| LZTR1 | Leucine-zipper-like transcriptional regulator 1 | 6xKelch + BTB/POZ; almost certainly NOT a transcription factor | Ubiquitous, golgi |  | Nacak et al^6^ |  |
| THAP7 | Thap (thanatos-associated) domain containing protein 7 | Zinc finger, proline-rich, coiled coil; binds to NcoR, HDAC3 and DNA |  |  | Macfarlan et al^7^ | ? - transcriptional repressor |
| MGC16703 | Tubulin pseudogene |  |  |  |  | Pseudogene |
| P2RXL1 | P2X purinoceptor 2 | ATP-gated cation channel (ionotropic receptor); other family members involved in pain, inflammation, neurotransmission. | CNS + others |  | Khakh et al^8^ |  |
| SLC7A4 | Cationic amino acid transporter | Transports cationic amino acid, or related small molecule (by Na+ exchange) - activity yet to be demonstrated. | Brain, testis, placenta |  | Sperandeo et al^9^ |  |
| LOC400891 | RNasin-like | LRR-containing, gene structure unclear |  |  |  |  |
| FLJ42953 | BCR-like Rho-GAP | Activator of Rho-GTPases |  |  |  |  |
| GGT-2  **LCR4** | Gamma-glutamyltransferase | Transfer of glutamyl moiety from glutathione to acceptors (metabolism of glutathione, synthesis of leukotrienes |  |  | Heisterkampet al^10^ | Several copies on chr22, including main gene GGT1. Primate-specific duplication. |
| RBP3.2 | RIMS binding protein 3B | Coiled coils, SH3, FNIII domains. Link between Ca2+ channels and synaptic vesicle tethering apparatus | Ubiquitous (unlike brain-specific RBP1 & 2) |  | Mittelstaedt et al^11^ | Three copies, all on chr22; two in this deletion |
| HIC2/HRG22 | Hypermethylated in cancer 2 | BTB/POZ, 5x zinc fingers (C2H2); paralogue of tumour suppressor HIC1. Probable recruiter of NCoR. Transcriptional repressor? | Widespread | Possible tumour suppressor? | Deltour et al^12^ |  |
| LOC220686/PI4KAP2 | PtdIns 4-kinase alpha-like | Phosphorylates lipid inositol headgroups |  |  |  | Pseudogene, related to PIKA4CA above |
| RBP3.3 | RIMS binding protein 3C | Coiled coils, SH3, FNIII domains. Link between Ca2+ channels and synaptic vesicle tethering apparatus | Ubiquitous (unlike brain-specific RBP1 & 2) |  | Mittelstaedt et al^11^ | Three copies, all on chr22; two in this deletion |
| UBE2L3/UbcH7 | Ubiquitin-conjugating enzyme E2 L3 | Ubiquitinates many targets, including p53 | Ubiquitous | Mouse mutant (proviral insertion with only 30% expression) gives retarded prenatal growth due to defective placenta | Harbers et al^13^ | Not obvious - no reported heterozygous or cardiac phenotype |
| LOC150223 | UPF0249 protein ydjC homolog | Unknown homologue of bacterial flagellar proteins - structural similarity to peptidoglycan NAG deacetylase |  |  | Imagawa et al^14^ |  |
| CCDC116 | Coiled coil domain containing 116 | Only one small coiled coil in 600-aa protein, no other domains. | very few ESTs, all in brain |  |  |  |
| SDF2L1 | Stromal cell factor 2-like protein 1/PWP1-interacting protein 8 | ER-targetted, MIR domain (ligand transferase?), possibly involved in O-glycosylation. Stress-induced. | Widespread, high in liver, low in heart (ER) |  | Fukuda et al^15^ |  |
| mir-301b | MicroRNA, unknown target | Downregulation of translation of target transcript (unknown) | ? |  | Post et al^16^ |  |
| mir-130b | MicroRNA, unknown target | Downregulation of translation of target transcript (unknown) | ? |  | Houbaviy et al^17^ |  |
| PPIL2 | Peptidyl prolyl cis-trans isomerase-like 2 (cyclophilin/rotamase), CyP-60 | Protein folding, either just post-translation (CD147), after denaturation, or as part of signalling process | Widespread, high in liver, testis, thymus | Potential modifier of Alzheimer's (upstream of BACE) | Wang et al^18^ |  |
| YPEL1 | Yippee-like 1 | One of 5 human proteins, homol to fly Yippee. Localises to centrosome and mitotic apparatus | Testis, foetal brain, foetal lung |  | Hosono et al^19^ |  |
| MAPK1 | Mitogen-activated protein kinase 1 (ERK2) | Part of kinase cascade by which mitogens regulate cell cycle progression | Ubiquitous | KO Mouse -/- dies in utero from poor extraembryonic tissue development. Also fewer +/- than expected, but those born are normal. | Saba-El-Leile et al^20^ | Not obvious - no reported heterozygous or cardiac phenotype |
| PPM1F | Protein phosphatase 1F (POPX2, hFEM-2) | Dephosphorylates PAK and CamKII, induces apoptosis |  |  | Koh et al^21^ |  |
| TOP3B | Topoisomerase 3 beta | Allows resolution of complex DNA topology. |  | KO mice develop normally but have short lifespan, accumulate aneuploidies, and die with lymphatic & renal problems. | Kwan et al^22^ | Not obvious - no reported heterozygous or cardiac phenotype |

Table 2: Genes in the deleted region.

1. Law RH, Zhang Q, McGowan S*, et al.* An overview of the serpin superfamily. *Genome Biol* 2006;**7**(5):216.

2. Sprecher E, Ishida-Yamamoto A, Mizrahi-Koren M*, et al.* A mutation in SNAP29, coding for a SNARE protein involved in intracellular trafficking, causes a novel neurocutaneous syndrome characterized by cerebral dysgenesis, neuropathy, ichthyosis, and palmoplantar keratoderma. *Am J Hum Genet* 2005;**77**(2):242-51.

3. Guris DL, Duester G, Papaioannou VE*, et al.* Dose-dependent interaction of Tbx1 and Crkl and locally aberrant RA signaling in a model of del22q11 syndrome. *Dev Cell* 2006;**10**(1):81-92.

4. Guris DL, Fantes J, Tara D*, et al.* Mice lacking the homologue of the human 22q11.2 gene CRKL phenocopy neurocristopathies of DiGeorge syndrome. *Nat Genet* 2001;**27**(3):293-8.

5. Xie Q, Lin T, Zhang Y*, et al.* Molecular cloning and characterization of a human AIF-like gene with ability to induce apoptosis. *J Biol Chem* 2005;**280**(20):19673-81.

6. Nacak TG, Leptien K, Fellner D*, et al.* The BTB-kelch protein LZTR-1 is a novel Golgi protein that is degraded upon induction of apoptosis. *J Biol Chem* 2006;**281**(8):5065-71.

7. Macfarlan T, Kutney S, Altman B*, et al.* Human THAP7 is a chromatin-associated, histone tail-binding protein that represses transcription via recruitment of HDAC3 and nuclear hormone receptor corepressor. *J Biol Chem* 2005;**280**(8):7346-58.

8. Khakh BS, North RA. P2X receptors as cell-surface ATP sensors in health and disease. *Nature* 2006;**442**(7102):527-32.

9. Sperandeo MP, Borsani G, Incerti B*, et al.* The gene encoding a cationic amino acid transporter (SLC7A4) maps to the region deleted in the velocardiofacial syndrome. *Genomics* 1998;**49**(2):230-6.

10. Heisterkamp N, Groffen J, Warburton D*, et al.* The human gamma-glutamyltransferase gene family. *Hum Genet* 2008;**123**(4):321-32.

11. Mittelstaedt T, Schoch S. Structure and evolution of RIM-BP genes: identification of a novel family member. *Gene* 2007;**403**(1-2):70-9.

12. Deltour S, Pinte S, Guerardel C*, et al.* Characterization of HRG22, a human homologue of the putative tumor suppressor gene HIC1. *Biochem Biophys Res Commun* 2001;**287**(2):427-34.

13. Harbers K, Muller U, Grams A*, et al.* Provirus integration into a gene encoding a ubiquitin-conjugating enzyme results in a placental defect and embryonic lethality. *Proc Natl Acad Sci U S A* 1996;**93**(22):12412-7.

14. Imagawa T, Iino H, Kanagawa M*, et al.* Crystal structure of the YdjC-family protein TTHB029 from Thermus thermophilus HB8: structural relationship with peptidoglycan N-acetylglucosamine deacetylase. *Biochem Biophys Res Commun* 2008;**367**(3):535-41.

15. Fukuda S, Sumii M, Masuda Y*, et al.* Murine and human SDF2L1 is an endoplasmic reticulum stress-inducible gene and encodes a new member of the Pmt/rt protein family. *Biochem Biophys Res Commun* 2001;**280**(1):407-14.

16. Post MJ, te Biesebeek JD, Wemer J*, et al.* Adenosine enhances antigen-induced bronchoconstriction and histamine release in rat isolated lungs. *Agents Actions* 1990;**30**(1-2):30-3.

17. Houbaviy HB, Murray MF, Sharp PA. Embryonic stem cell-specific MicroRNAs. *Dev Cell* 2003;**5**(2):351-8.

18. Wang BB, Hayenga KJ, Payan DG*, et al.* Identification of a nuclear-specific cyclophilin which interacts with the proteinase inhibitor eglin c. *Biochem J* 1996;**314 ( Pt 1)**:313-9.

19. Hosono K, Sasaki T, Minoshima S*, et al.* Identification and characterization of a novel gene family YPEL in a wide spectrum of eukaryotic species. *Gene* 2004;**340**(1):31-43.

20. Saba-El-Leil MK, Vella FD, Vernay B*, et al.* An essential function of the mitogen-activated protein kinase Erk2 in mouse trophoblast development. *EMBO Rep* 2003;**4**(10):964-8.

21. Koh CG, Tan EJ, Manser E*, et al.* The p21-activated kinase PAK is negatively regulated by POPX1 and POPX2, a pair of serine/threonine phosphatases of the PP2C family. *Curr Biol* 2002;**12**(4):317-21.

22. Kwan KY, Moens PB, Wang JC. Infertility and aneuploidy in mice lacking a type IA DNA topoisomerase III beta. *Proc Natl Acad Sci U S A* 2003;**100**(5):2526-31.
